# Plant Spices as a Source of Antimicrobial Synergic Molecules to Treat Bacterial and Viral Co-Infections

**DOI:** 10.3390/molecules27238210

**Published:** 2022-11-25

**Authors:** Nathália Barroso Almeida Duarte, Jacqueline Aparecida Takahashi

**Affiliations:** 1 Department of Food Science, Faculty of Pharmacy, Universidade Federal de Minas Gerais, Av. Antônio Carlos, 6627, Belo Horizonte CEP 31270-901, Brazil; 2 Chemistry Department, Universidade Federal de Minas Gerais, Av. Antônio Carlos, 6627, Belo Horizonte CEP 31270-901, Brazil

**Keywords:** bacterial resistance, combination therapy, COVID-19, medicinal plants, new drugs, synergism

## Abstract

The COVID-19 pandemic exposed the lack of antiviral agents available for human use, while the complexity of the physiological changes caused by coronavirus (SARS-CoV-2) imposed the prescription of multidrug pharmacotherapy to treat infected patients. In a significant number of cases, it was necessary to add antibiotics to the prescription to decrease the risk of co-infections, preventing the worsening of the patient’s condition. However, the precautionary use of antibiotics corroborated to increase bacterial resistance. Since the development of vaccines for COVID-19, the pandemic scenario has changed, but the development of new antiviral drugs is still a major challenge. Research for new drugs with synergistic activity against virus and resistant bacteria can produce drug leads to be used in the treatment of mild cases of COVID-19 and to fight other viruses and new viral diseases. Following the repurposing approach, plant spices have been searched for antiviral lead compounds, since the toxic effects of plants that are traditionally consumed are already known, speeding up the drug discovery process. The need for effective drugs in the context of viral diseases is discussed in this review, with special focus on plant-based spices with antiviral and antibiotic activity. The activity of plants against resistant bacteria, the diversity of the components present in plant extracts and the synergistic interaction of these metabolites and industrialized antibiotics are discussed, with the aim of contributing to the development of antiviral and antibiotic drugs. A literature search was performed in electronic databases such as Science Direct; SciELO (Scientific Electronic Library Online); LILACS (Latin American and Caribbean Literature on Health Sciences); Elsevier, SpringerLink; and Google Scholar, using the descriptors: antiviral plants, antibacterial plants, coronavirus treatment, morbidities and COVID-19, bacterial resistance, resistant antibiotics, hospital-acquired infections, spices of plant origin, coronaviruses and foods, spices with antiviral effect, drug prescriptions and COVID-19, and plant synergism. Articles published in English in the period from 2020 to 2022 and relevant to the topic were used as the main inclusion criteria.

## 1. Introduction

The approval of the first antiviral drug for human use (idoxuridine) occurred in 1963 and, despite all scientific and technological development, the list of antiviral drugs available as medicines is still insufficient [1]. Research in this area is necessary, especially because viruses use the host cells to multiply and antiviral substances usually cause multiple side effects [2]. Many viruses are endemic in various parts of the world, such as Ebola, HIV, hepatitis B, dengue, zika, flu, and, more recently, coronavirus (SARS-CoV-2). Vaccination reduced the spread of pandemic and endemic viral diseases, but a relevant number of individuals are still infected by viruses daily. Although antiviral drugs can be useful in the treatment of some viruses, the development of less toxic effective drugs is desirable, especially considering the 2019 coronavirus pandemic scenario, which exposed the lack of effective and safe antiviral drugs and the need for advances in research to develop new drugs with faster results and less side effects. Remdesivir prescribed to severely ill patients resulted in faster recovery; however, for higher effectiveness, anti-inflammatory or immunomodulators such as baricitinib, tocilizumab, and bamlanivimab must be co-administrated to target both viral proliferation and the hyperimmune response [3]. Remdesivir’s safety and scope of action is still the target of several clinical studies, although some side effects such as cardiotoxicity have already been reported in an in vitro human cardiac model. This side effect must be further evaluated to avoid fatalities and long-term sequelae, as cardiac-compromised individuals have a worse prognostic of COVID-19 [3]. Besides remdesivir, molnupiravir, fluvoxamine and Paxlovid are also oral antiviral drugs with a positive reduction in the mortality and hospitalization rates of COVID-19 patients, and they also feature good overall safety [4]. Molnupiravir has the advantage of being effective in five days and avoiding major events, as observed in clinical trials, although one study pointed out that this drug caused mutations. Molnupiravir is effective in the first days after the initial symptoms and is recommended by the OMS, but it is recommended to treat mild cases. Fluvoxamine’s mechanism of action has drawn attention, while Paxlovid, a SARS-CoV-2 protease inhibitor, was designed to work directly on the novel coronavirus-specific protease [4]. However, side effects and the need for broader coverage led to clinical studies aimed at repurposing several drugs as antivirals to fight COVID-19, but most of them failed in the clinic studies or showed inconsistent results.

In addition, the complexity of the physiological changes caused by coronavirus has created several challenges, and adjustments were added to the antiviral pharmacotherapy scenario at the beginning of the COVID-19 pandemic. The treatment of individuals hospitalized with COVID-19 and severe acute respiratory syndrome (SRAG) was unsuccessful with the single prescription of antiviral agents and, in most cases, the administration of a pool of medicines was required. Among these medicines, antibiotics were frequently prescribed to prevent coinfections. Due to the pandemic dimension, this huge increase in the use of antibiotics has contributed to the development of acquired bacterial resistance [5]. This resistance appears when bacteria are able to make changes in the active site targeted by the antibiotic, reducing the infiltration of the active compound in the bacterial cell or even releasing enzymes capable of causing structural degradation in the antibiotic structure [6]. By 2030, the economic reflection of antimicrobial resistance problems could lead to extreme poverty for up to 24 million people [7]. According to the UN ad hoc Group on Interagency Coordination on Antimicrobial Resistance, drug resistance could cause 10 million deaths per year [7]. In this scenario, the development of new antibacterial drugs, together with antiviral drugs, is a world priority, in order to address the still ongoing demand caused by COVID-19, but also to decrease the number of deaths associated with bacterial infections worldwide. New antibacterial and antiviral drugs are expected to cause less side effects and are intended for several groups of individuals that still cannot rely on safe medicines, such as patients with comorbidities or immunocompromised and pregnant women. In addition, new antimicrobials are expected to have mechanisms of action, therefore, making it possible to fight resistant bacteria and viruses. Moreover, low cost is extremely desirable, in order to increase access to the treatment.

Plants remain an interesting source of novel molecules due to their proven efficacy, adequate bioavailability and easy applicability in human consumption, as well as the positive history of plant products that have become a source of modern prescription medicines [1,8]. Artemisinin is one of the most recent and successful examples of natural products that have reached the international market as a finished pharmaceutical product. Artemisinin and its derivatives have been adopted by dozens of countries as a first-line anti-malarial drug, and it has a significative bulk export production. Among the advantages of artemisinin over other anti-malarial drugs, this natural product provides a rapid decrease in parasitemia and delays drug resistance in combination therapy [9]. The good outcomes of new medicines from plants in several fields has led to an intense search for new drug leads to combat the coronavirus and to treat illnesses associated to COVID-19 [10].

However, drug development from plant metabolites is a slow process, contrasting with the urgent demand for new antiviral and antibacterial agents. In addition, once a promising molecule is identified, toxicity screenings, formulation studies, bioavailability and, later, the scale up for pilot industrial production can be limiting factors for reaching pre-clinical and clinical phases [11]. A faster strategy, likely to be successful, is based on the research of plant species already consumed by humans (i.e., with known toxicity) and industrially produced (i.e., available on a large-scale basis and with industrial processing already established) [12]. Spices of vegetable origin are among the plant sources that fit into this profile. A huge number of spices produced on an industrial scale and which are safe for human consumption and used in different countries are described in the scientific literature as promising antibacterial and antiviral agents [13,14].

In this review, we initially present data demonstrating the need for new antiviral and antibiotic drugs, especially corroborated within the scenario of the recent COVID-19 pandemic. The second part discusses the potential of plants widely used as spices, with antibacterial, antiviral and related properties described in the literature, for the development of new antiviral and antibacterial drugs. The pharmacological potential of the secondary metabolites produced by these species, the synergic effects and the current research on antivirals are also discussed.

## 2. COVID 19: Context, Treatment and New Drugs Demand

### 2.1. COVID-19 Scenario

COVID-19 originated from the SARS-CoV-2 virus of the Coronaviridae family. The organization of SARS-CoV-2 has been associated with the presence of 16 non-structural and four structural proteins: peak (S), envelope (E), membrane (M) and nucleocapsid (N). The S protein binds to the ACE2 receptor, which is located on the host cell surface, and allows the insertion of RNA into the host cell cytoplasm. This interaction also requires the priming of the S protein that takes place when SARS-CoV-2 binds to the protease. As a result, the binding of SARS-CoV-2 to the ace2 receptor allows the virus to enter the host cell, mainly by endocytosis, where it initiates a deregulated immune response, resulting in acute injuries [15].

The most common clinical symptoms in individuals affected by COVID-19 disease are fever (98% of cases), followed by cough (76%), dyspnea (55%) and myalgia or fatigue (44%). The recurrent laboratory characteristic among those infected with the virus is leukopenia (25% of cases), a worrying condition because the reduction in white blood cells in the bloodstream decreases the body’s ability to fight infections, resulting in immunity output [16]. The clinical symptoms of COVID-19 were classified as mild, moderate or severe, with the need to prescribe antibiotics predominantly for severe and moderate conditions [17]. Dyspnea is the main motivation for intubation, a recurrent scenario related to the clinical complications caused by COVID-19. In this process, a cannula is positioned through the gloat to the region of the arytenoid cartilages vocal process. These arytenoids are coated with a thin layer of the mucosa perichondrium, and intubation makes these cartilages susceptible to trauma, excessive cough, upper respiratory tract-related infections, and pneumonia, clinical conditions that also require the prescription of antibiotics [18].

Comorbidities and advanced age were earlier associated with hospitalization and severe symptoms among patients with COVID-19. The immune debit caused by some comorbidities decreases the individual’s resistance to SARS-CoV-2, making these individuals prone to more severe infections than those without comorbidities. These conditions predispose to the development of bacterial infections, increasing the possibility of coinfection and hindering the convalescence of patients [19]. A study with 315 patients who tested positive for COVID-19 revealed that 95 (30%) had comorbidities such as chronic obstructive pulmonary disease, hypertension, cardiovascular alterations, diabetes mellitus, chronic kidney disease and cancer. Among these 95 individuals, 69 presented superinfections caused by bacteria, impacting the length of hospitalization (30 days), while the hospitalization period of those that did not develop this condition was 11 days [20].

COVID-19-related morbidities fall under the class of chronic non-communicable diseases (NCCD). NCCDs are responsible for 70% of deaths worldwide, of which 80% occur in low- and middle-income countries (72.6% in Brazil), and the need to prescribe antibiotics for patients in this group greatly increased with the COVID-19 pandemic [21]. In a study conducted in China, it was reported that among a group of 856 patients diagnosed with COVID-19, approximately 30–50% had one or more comorbidities, mainly hypertension (30–50%), diabetes (8–20%), cardiovascular disease (5–20%), chronic liver disease (1–5%) and chronic kidney disease (1- 4%). The study proved that the higher the number of comorbidities, the greater the risk of developing severe conditions and bacterial infections [22]. Among the factors that aggravated the number of people affected by COVID-19, physical inactivity, alcohol abuse, inadequate diet and smoking are frequently cited [21].

Clinical trials have been conducted on dozens of vaccines worldwide, and some of them have received emergency authorization to be used for active and preventive immunization against COVID-19. Some vaccines act by stimulating the production of neutralizing antibodies responsible for protecting the immune system [23]. Immunization through the vaccine has had a positive effect, but the disease is far from being controlled, especially by the emergence of more lethal or more contagious variants [24].

### 2.2. Combination Pharmacotherapy for Treatment of Patients with COVID-19

Effective drug therapies for the treatment of people infected with the SARS-CoV-2 virus have been discussed since the beginning of the pandemic. Combinations of drugs with active ingredients already registered for the treatment of other diseases with symptoms correlated with COVID-19 have been widely explored [25]. This approach expedites the release of drugs when early-stage clinical trials have already been conducted and the drugs are already supplied on the market [26]. However, there is still concern about patient safety, due to the adverse effects associated with the combined use of these drugs, since most of them have already caused side effects when used individually [27].

A main target of drugs within the scope of COVID-19 is the SARS-CoV-2 protein, and the proposed therapies act on viral enzymes or functional proteins, RNA synthesis and replication, blocking the binding of the virus to human cell receptors. They target structural proteins, restore host innate immunity, inhibit virulence factor, or act on host-specific receptors, thus, preventing viral entry [4,28,29].

In numerous completed or ongoing clinical trials, various antiviral and immunomodulatory molecules have been administered to patients with severe COVID-19. In an extensive review, Salasc and colleagues [27] described the combination of treatments used worldwide to combat COVID-19 through randomized clinical trials, such as remdesivir plus baricitinib, lopinavir-ritonavir plus ribavirin and interferon beta-1a (IFNb-1b), as well as dexamethasone together with azithromycin or remdesivir. Remdesivir, lopinavir/ritonavir, and favipiravir are capable of inhibiting viral enzymes or functional proteins, RNA synthesis and replication, thus, exhibiting an effect against SARS-CoV-2 [30].

**Figure 1:**
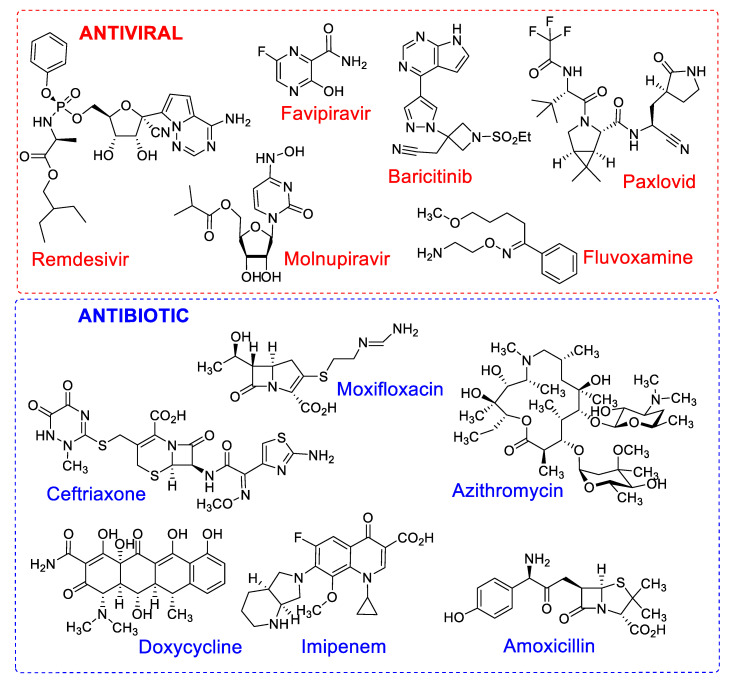
Chemical structures of antiviral and antibiotic drugs with different mechanisms of action used in mono or combination therapy in the treatment of patients with COVID-19.

Some medicines of the corticosteroid class—including cortisone, prednisone and methylprednisolone—were also implemented in combination therapies as steroidal anti-inflammatory drugs, as well as prescribed antibiotics, for example, azithromycin, ceftriaxone, moxifloxacin and imipenem [31]. The chemical structures of the main antiviral and antibiotic drugs can be found in Figure 1. The synergistic effect of combination therapies delays the onset of drug resistance, due to the additive effect, increasing the safety and efficacy of the treatment [32].

### 2.3. Prescription of Antibiotics for Patients with COVID-19 and Bacterial Resistance

**Figure 2:**
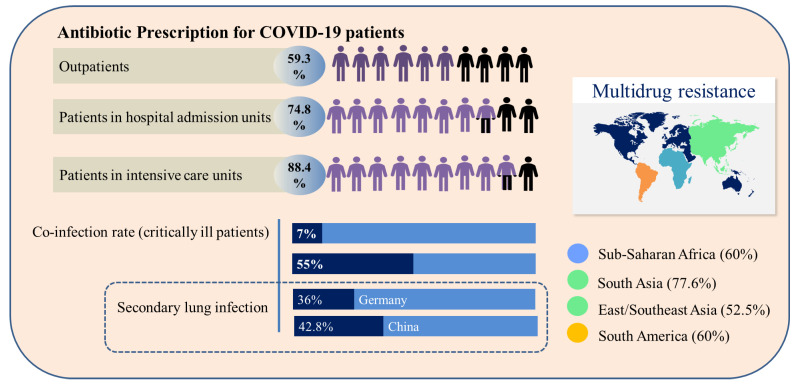
Comparative overview of antibiotic prescription, co-infection and secondary infection profile and geographic incidence of multidrug resistance related to COVID-19 patients.

Currently, there are multiple demands for new antibiotics, some of which are due to the increasing number of serious post-surgery infections caused by resistant bacteria [33]. WHO guidelines did not indicate the prescription of antibiotics for patients with suspected or confirmed COVID-19 with mild symptomatology, nor for those with low suspicion of bacterial infection. However, in severe cases where diagnosis, treatment and prognosis are difficult, the administration of antibiotics is necessary to increase the chance of patients’ survival. This precautionary measure contributes to the emergence of multidrug-resistant strains and to the reduction in the efficacy of the antibiotics currently available [34]. As the patient’s condition becomes severe, the antibiotic prescription increases, as shown in Figure 2, and is 64.4% higher for patients requiring mechanical ventilation, as bacterial and fungal coinfections are common in critically ill patients [33]. According to Su et al. [35], 50% of deaths in severe patients with COVID-19 occur in patients with secondary infections. Specifically, in patients with COVID-19, the bacterial co-infection rate reaches 7.7% of cases, and it is mainly related to *Acinetobacter baumannii*, *Escherichia coli*, *Pseudomonas aeruginosa* and *Enterococcus* sp. Patients usually receive broad-spectrum antibiotics such as azithromycin, ceftriaxone, imipenem and moxifloxacin. However, resistant bacteria are a huge problem. Imipenem, a broad-spectrum β-lactam antibiotic, significantly reduces inflammatory cytokines, with better results for the treatment of COVID-19 patients with nosocomial bacterial infections. Resistance to carbapenem, an antibiotic of the class of imipenem, has been reported in patients co-infected with *A. baumannii* (55.6%), imposing treatment difficulties and increasing the likelihood of septic shock [36].

The incidence of secondary lung infections caused by bacteria in COVID-19 patients reached 36% in Germany and 42.8% in China [37]. The geographic distribution of multidrug-resistant bacteria which are not affected by the action of conventional antibiotics is presented in Figure 2 [38]. Infections by multidrug-resistant bacteria in hospital environments are serious, due to the concentration of individuals with fragile health that are more susceptible to infections caused by opportunistic pathogens. The main pathogens related to these infections are identified with the acronym ESKAPE (*Enterococcus faecium*, *Staphylococcus aureus*, *Klebsiella pneumoniae*, *A. baumannii*, *P. aeruginosa* and *Enterobacter species*), which is characterized by bacteria capable of “escaping” the actions of antibiotics [36]. Beyond the issues associated with the mortality derived from ESKAPE bacteria, bacterial triggers associated with these organisms are supposed to participate in the manifestation of some autoimmune diseases, such as rheumatoid arthritis and multiple sclerosis [39,40,41].

### 2.4. Medicinal and Spice Plants with Antibiotic Activity and Their Synergistic Effects with Industrialized Antibiotics

Industrialized antibiotic accessibility is a huge problem in many parts of the world, due to their cost and the need for a strict uptake routine. Other issues such as stability also contribute to this scenario. To overcome stability issues, some antibiotics must be commercialized as dry powders to ensure activity and extend the shelf life, imposing a step of rehydration for their reconstitution that may become a further problem under several circumstances [42,43]. These issues can be minimized with the use of medicinal plants. Plants with antibiotic power can be used in natura or after simple processing steps, such as extraction, pulverization and drying, or boiling, without a loss of the pharmacological effects [44]. In addition, using the whole plants or their parts instead of commercial antibiotics has the advantage of reduced cost, making this alternative treatment more accessible for the population unable to use prescription antibiotics. A review on the world trends in medicinal plants research, published in 2020 [45], showed that over 110,000 studies on this theme were published between 1960 and 2019, most of which were classified within the scope of pharmacology, toxicology and pharmaceutics. It was estimated that 10% of all vascular plants are used due to their medicinal properties aiming at preventive, control and curative activities [46].

The mechanism of plant extracts’ antibacterial action depends on several factors, such as the characteristics of the target bacterium, extract composition, and the chemical features of the phytoconstituents. In general, this mechanism usually involves disruption and lysis of the microorganism’s cell wall, the release of cellular content, protein binding domain disruption, inhibition of microbial DNA replication and nucleic acid transcription, inhibition of the biosynthesis of compounds toxic to the host and other effects that lead to cell death or a decrease in deleterious effects [47]. The treatment of infections with plant extracts has not been suggested as a major factor in the development of bacterial resistance [48,49], and a number of plant extracts are active against resistant bacteria, as shown in Table 1. Among the plants cited in Table 1, *C. englerianum*, *E. depauperate*, *M. chamomilla*, *T. zygis*, and *T. willdenowii* showed high IZ values, therefore, pointing to the inhibition of resistant bacterial strains. In some studies, minimum inhibitory concentration (MIC) determination provided additional details, such as selectivity [50,51,52,53,54,55], and may be preferable to determine the full antimicrobial activity [56]. For example, IZ values observed for *A. aspera* in the presence of different resistant strains do not vary significantly (6.0–6.3 mm), but MIC data demonstrated that this plant is more active against MRSA and MRKP in relation to MrPA [50]. In another study, *L. inermis* presented higher IZ values for MRSA and MRPA, suggesting that these bacteria are more susceptible to the extract than MRKP, which was confirmed by MIC data [50].

Activity against resistant bacteria through new strategies has been described for plant extracts, such as the quorum-sense mechanism, which is considered a promising alternative to antibiotics. This mechanism, mediated by signal molecules, can be applied to multiple pathogenic bacteria and is based on the action of auto-inducers’ self-excreted molecules capable of regulating the expression of virulence genes [58]. Overall, quorum-sensing acts in the host defense, controlling biofilm formation and the production of compounds that are toxic to the bacteria. The early inhibition of biofilm formation was reported over *S. aureus* and *S. pyogenes*. The restriction of van der Walls and electrotactic interactions, avoiding the attachment of pathogenic bacterial cells, was a mechanism of action that explains the decrease in infection [49].

Technological development has been successfully used to improve antibacterial activity, as in the development of clusters of natural products with silver ions. The clusters attach to the surface of the bacterial cell wall, damaging the cell surface. Moreover, silver ions disable the microbial growth process, as shown for the *A. catechu*-Ag cluster [58].

Complementing the new mechanisms of action, other major benefits of using the whole plant concern the well-known synergic and/or complementary effects of the different metabolites present in plants/plant extracts. In cumin, the antimicrobial activity is related to the synergy among terpenes and some minor constituents such as limonene, eugenol and pinene (Bazaka et al., 2015). The isolation of the bioactive metabolites may be unnecessary, since the phytoconstituents can be less active once administered alone, due to the lack of synergic compounds [41,64]. In this way, the concomitant use of industrialized antibiotics and bioactive plant extracts—the so-called antibiotic-adjunct combination strategy—has been proposed to overcome problems related to resistant bacteria [65]. The efficacy of combination therapy can be exemplified by the use of penicillinase and β-lactamase inhibitors as co-drugs to overcome bacterial resistance to some antibiotics. Combinatorial plant-drug therapies have already been indicated to treat viral infectious diseases, such as AIDS [66]. Even plant-drug combinations lacking synergic effects do not interfere in the individual activity of the antibiotic, which is highly encouraging for research and the application of combinational therapies [30,41].

The synergism between antibiotics (ampicillin, penicillin, tetracycline, methicillin, etc.) and several plant extracts (green tea, khat, pomegranate, basil, lemon balm, grape pomace and oregano), plant metabolites (curcumin, epigallocatechin-gallate, flavonoids, alkaloids and terpenes), as well as essential oils is widely described, and some examples are shown in Table 2. In general, synergy can be achieved by the blockage of multidrug resistance pumps, consequently facilitating the entrance and traffic of antibacterial agents into the bacterial cell [8]. Therefore, combinational synergistic approaches may be a good tool to combat resistant bacterial strains [41]. Simple and general methods exist that are capable of evaluating the synergistic effect of drug combination, enabling measurement of the qualitative and quantitative physiological effects of two or more drugs [67].

As observed in Table 2, the association of plants with antibiotics lead to synergistic effects; this is important, as it allows the administration of lower doses of the industrialized antibiotic, with the same or even higher antibiotic effects, contributing to postponing the phenomenon of resistance. The inhibition of efflux pumps by some phytochemicals consequently avoids bacterial exposure to sub-therapeutic concentrations of antibiotics, which is one of the causes of acquired resistance [71]. Plant metabolites that favor morphological disruptions on the bacteria cell wall help to decrease the number of functional bacterial cells to be attacked by the antibiotic, as postulated for the synergistic interaction of thymol–ciprofloxacin [74]. The antimicrobial activity mechanism of thymol has been linked to the presence of free hydroxyl groups that are involved in membrane depolarization [74]. The action of this phytochemical plays an important role in potentializing the antibiotic effect of commercial drugs such as ciprofloxacin. In some examples, plant metabolites increase their outer membrane permeability to allow antibiotic activity. Flow cytometry was used to show that *V. diospyroides’* synergistic action in combination with ampicillin is the result of cell membrane granularity disruption [76]. Plants can act directly in specific intracellular enzymes or deactivate the production of enzymes produced by bacteria to degrade the antibiotic chemical structure, therefore, helping the antibiotic to enter the bacterium cell to exercise its activity [71]. The synergic multi-target action of phytochemicals can also influence the production of enzymes necessary for endogenous energy production and protein synthesis.

### 2.5. Potential of Plant Spices with Antibiotic Activity as Antiviral Agents

Plants used as spices have some interesting particularities that can be explored in the search for natural remedies to treat patients requiring the simultaneous use of antibacterial and antiviral agents. First, the widespread consumption of plant-derived spices over time has demonstrated their safety for human consumption [78]. Second, spices are likely composed of stable compounds, as they are frequently used in hot dishes, and concomitantly with acid compounds in food preparation, such as vinegar and lemon. Third, the use of such spices as adjuncts, including in COVID-19 prevention or treatment, would rely on the patient’s consent, as these plants are already traditionally consumed [79]. Moreover, these plants are usually commercialized as finely sprayed powders with a long shelf-life, facilitating the development of multiple formulations. The safety, authentication and traceability of condiments are well studied, and effective non-destructive vibrational and atomic spectral analytical techniques are available for quality control and meet the increasing industrial demand [80,81]. The global condiment market, estimated to be worth USD 76.6B by 2020, is projected to reach USD 103.7B by 2026 [82]. China, Turkey, Vietnam and Indonesia are the markets leaders in the growth of this sector and, in the coming years, they should be influenced by trends related to health, gourmet flavors and ethical values.

**Figure 3:**
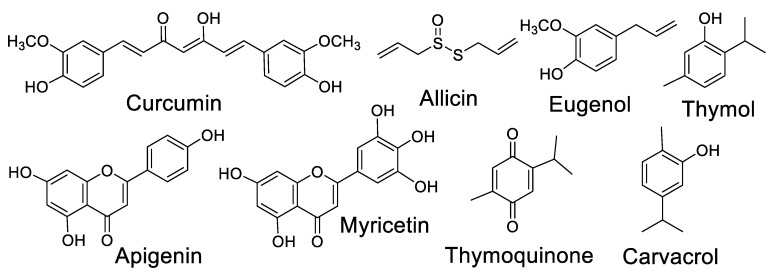
Chemical structures of relevant antiviral natural products in the context of COVID-19.

Several plant spices with antibiotic activity have gained attention as antiviral agents, especially against SARS-CoV-2. The consumption of plant spices with antibiotic and antiviral properties by patients with mild and moderate cases of viral infections deserve deeper studies, as they could assist in avoiding and combating concomitant infections. Some secondary metabolites present in these plants have been proven as potent antiviral compounds. The chemical structures of some relevant antiviral natural products in the context of COVID-19 are presented in Figure 3.

**Figure 4:**
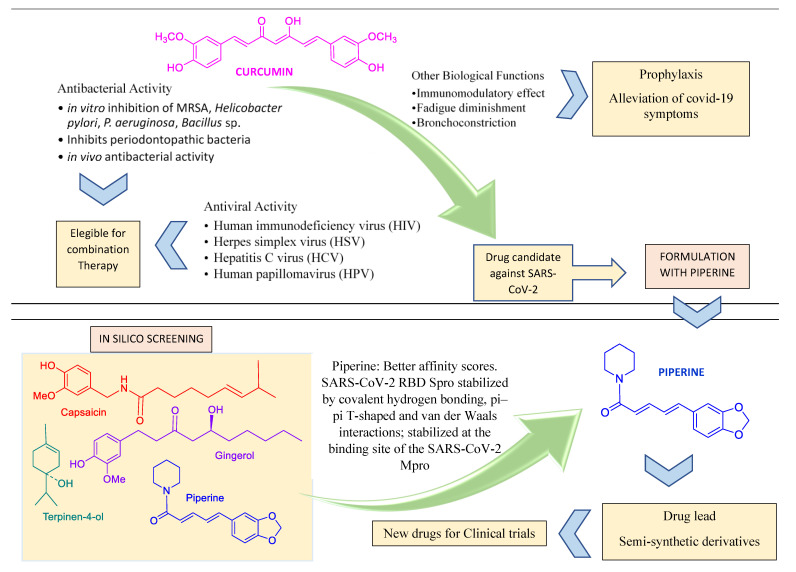
Curcumin and piperine features compatible with their use in combined pharmacotheraphy.

Curcumin, a natural component of *Curcuma longa*, is one of the most prominent examples of a worldwide consumed condiment which has both antibacterial [83] and antiviral properties [84] (Figure 4). Curcumin diminishes fatigue and bronchoconstriction, helping to alleviate the symptoms of COVID-19 [85]. The antiviral action mechanism of curcumin was recently reviewed [84]. The administration of curcumin in patients with COVID-19 on a formulation containing piperine (a compound that promotes the absorption of curcumin) improved some symptoms, compared to the control group, on a randomized clinical trial [86]. Curcumin is also suggested as a promising prophylactic candidate for the treatment of COVID-19 and is a target of other studies towards the development of nano formulations and supplements [87,88,89,90,91,92,93,94].

Allicin [95,96], eugenol [97,98], thymoquinone [99], apigenin [100,101], carvacrol [102,103] and thymol [104] are some of the most studied metabolites of spice plants with antiviral effects and consequent applications in the efforts to combat COVID-19 (Table 3).

Garlic is a condiment native to Asia and has been used as a medicine since ancient times, as in Egypt, where it was consumed during the construction of the pyramids. These and other historical features, as well as their antibacterial properties—especially those related to the organosulfur metabolites of garlic—were recently reviewed [105]. Garlic also has immunomodulatory, anti-inflammatory, anticancer, antitumor, antidiabetic and cardioprotective effects. Allicin, the major component of raw garlic, is a broad-spectrum antimicrobial agent. The inhibition of specific bacteria by garlic has been studied, as in the case of *Streptococcus agalactiae* (Group B *Streptococcus*), an invasive bacterium suggested to be the major cause of neonatal morbidity and mortality during the first weeks of life [106]. Garlic has been reported as active against over twenty viruses, including adenovirus, herpes simplex virus, influenza A virus and human immunodeficiency virus [107]. In silico studies targeting host receptor angiotensin-converting enzyme 2 (ACE2) protein related to coronavirus resistance showed strong interactions of garlic essential oils with the amino acids of the ACE2 protein and the main protease of SARS-CoV-2 (PDB6LU7). In addition, synergistic interactions of components were observed, resulting in good inhibition of the ACE2 and PDB6LU7 proteins [108]. On a broad docking screening with 75 metabolites present in traditional Indian spices, one of the highlights was the flavonoid myricetin, a metabolite produced by *A. sativum*. The activity of myricetin was more pronounced against main proteases (M^pro^) than against SARS-CoV-2 spike proteins (SP) [109]. Pre-clinical and clinical studies of the effect of garlic on viral infections, including those caused by SARS-CoV, have shown the effectiveness of garlic’s antiviral capacity by means of several mechanisms, such as downregulation of the extracellular-signal-regulated kinase (ERK) and mitogen-activated protein kinase (MAPK) signaling pathway, the blockage of viral entry into host cells, and inhibition of viral RNA polymerase [107].

Another spice, *N. sativa* (*black cumin*)—a healing herb of the Ranunculaceae family and much appreciated in North Africa—is traditionally used to treat various diseases including hypertension, asthma, inflammation, diabetes, cough, headache, bronchitis, eczema, dizziness and fever. Black cumin’s pharmacological properties, such as immunomodulatory, anti-inflammatory, antimicrobial, antioxidant and anticancer activity are described [110], in addition to the inhibition of SARS-CoV-2 [111,112]. In a broader context, the immunomodulatory components present in traditional plants, such as basil (*O. sanctum*) and cinnamon (*C. verum*), can potentiate the effect of antibiotics when administered together (Table 3). Basil has an anti-inflammatory and antimicrobial agent named eugenol, while cinnamon is used in traditional medicine for various lung-related disorders, including pneumonia, infectious diseases and pleural effusion [113]. Other essential oils from spices have also been reported as antimicrobial agents [114].

**Table 3:** Plant spices and condiments and their antiviral profile.

Scientific Name [Popular Name]	Main Component	Antiviral Effects/COVID-19 Applications	Reference
*C. longa* [Turmeric]	Curcumin	Attenuation of poly(I:C)-induced immune and inflammatory responses by inhibiting the TLR3/TBK1/IFN-β cascade	[90]
		Enhancement of oral drug delivery system (Labrasol^®^ and tween 80 bicelles)	[91]
		Molecular docking studies showed reliable ADME profile	[92]
		Analogues as dual inhibitor of SARS-CoV-2	[93]
		Development of nanoformulations	[87,94]
*Allium sativum* [Garlic]	Allicin	Suppresses production and secretion of pro-inflammatory cytokines and stimulates of immune system cells (NK, lymphocytes, eosinophils and macrophages)	[95]
		Suppression of pro-inflammatory cytokines TNF-α and CRP	[96]
*Cinnamomum verum* [Dalchini]	Eugenol	Inhibition of specific immune responses to allergens, reduces side effects of some anti-inflammatory drugs, antioxidant properties	[97]
		Increases the bioavailability of antiviral drug saquinavir	[98]
*Nigella sativa* [Black cumin]	Thymoquinone	Inhibitory effects on viral spike protein with cellular angiotensin-converting enzyme 2 (ACE2)	[99]
		Inhibition of RdRp of SARS-CoV-2, especially α-hederin; ongoing drug development strategy against SARS-CoV-2	[99]
*O. basilicum* [Basil]	Apigenin	The phytoconstituents vicenin, sorientin and ursolic acid inhibit SARS-CoV-2 M^pro^	[100]
		Development of gellan gum hydrogel with basil oil nanoemulsion	[101]
*O. vulgare* [Oregano]	Carvacrol	Inhibition of viral replication and activity of SARS-CoV-2 3CL^PRO^	[102]
		Potent inhibition of SARS-CoV-2 replication (modeling studies)	[103]
*Thymus vulgaris* [Thyme]	Thymol	Inhibits the viral spike protein, preventing SARS-CoV-2 entry	[103]
		Essential oils induce cytopathogenic effect against SARS-CoV in Vero-E6 cells	[104]

The therapeutic properties of *O. vulgare*, popularly known as oregano, are associated with terpenes and flavonoids. Antiviral studies demonstrated strong inhibition of viral replication and SARS-CoV-2 3CL^pro^ activity, while in molecular modeling assays, terpe-noids were highlighted as potent inhibitors of SARS-CoV-2 replication [102,103]. Thyme (*T. vulgaris*) is also reported to be active against fungi, viruses and bacteria. It is used as a flavoring agent for cheese and beverages, and in traditional medicine to cure melancholic conditions, skin and respiratory lesions [115]. Thyme essential oil has been shown to be effective against several RNA viruses, including coronaviruses, and it induces a cytopathogenic effect against SARS-CoV in Vero-E6 cells [100,104].

**Figure 5:**
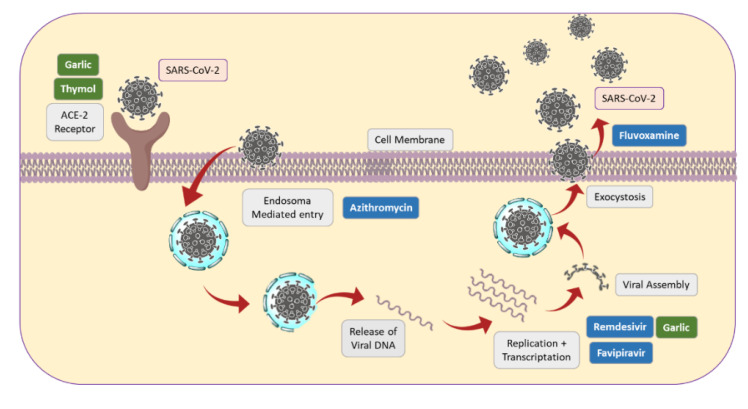
Some key steps of SARS-CoV-2 replication, together with action sites of some drugs and spices.

Figure 5 shows a scheme on some key steps in SARS-CoV-2 penetration, replication and exit, as well as some medicines and spices that act in several points.

### 2.6. Recent Research in Spice-Derived Metabolites in COVID-19 Context

The recent literature shows a huge number of studies on traditional plant spices to enable the search for drugs that protect against COVID-19 and other possible viral pandemics. It is unnecessary to highlight the need to support these studies to avoid, as much as possible, other viral pandemics. Some of these studies and related review papers are listed in Table 4.

These review papers demonstrate that endophyte fungi as a source of new drug leads to treat individuals with COVID-19 is a major research theme all over the world [116,117]. The research is mainly associated with host plants that have key biological activities, including species already used to fight COVID-19 symptoms [121,127]. Some works searched metabolites to be used in the prevention of viral infections, by means of strengthening the immune system [122,123]. Pure natural components of plant spices have also been reviewed [124], and several other approaches have been developed. The number of studies listed in Table 4, and the information gathered in this review clearly shows the potential of plants, and specifically, plant spices, as antibacterial and antiviral agents, alone or associated with industrialized medicines. Although it is important to study new plants to discover novel metabolites, there is much that can be done, and more quickly, using the scientific knowledge already available in the literature about specific plants. Although plant metabolites have a natural origin, problems such as the development of allergies, the use of ineffective dosages, toxicity caused by over-dosage, the form of administration, and standardization must be considered at some point. In addition, it must be considered that some spices have high added value and may undergo adulteration during marketing by adding other plants, flour, etc., diluting the active ingredient content.

However, the volume of scientific information proving the pharmacological attributes of hundreds of plants is highly disproportionate to their use as medicines, especially in Western countries. The pharmaceutical industry is expected to invest further in this area to produce more affordable and sustainable antiviral and antibacterial medicines.

## 3. Conclusions

The development of combination therapies that include antivirals, antibiotics and other drugs was very useful for the treatment of patients infected with coronavirus during the pandemic. However, the development of new antiviral drugs with fewer side effects is still necessary, in order to be prepared against other viruses that may emerge, as well as those circulating endemically in several countries. Among the various problems inherited by the current pandemic, we pointed to the increase in resistant bacterial strains, as the result of the prescription of antibiotics for patients with COVID-19 as a prophylactic measure. Medicinal plants are generally safe and highly accessible for consumption and have been used in many countries to prevent or cure the symptoms of COVID-19, although there is no consensus on the effectiveness of bioactive compounds in low concentrations. Among medicinal plants, plant spices frequently contain components with antiviral and antimicrobial activities, and the synergic interaction of multiple metabolites can improve the biological activity. The industrial availability of many plant spices increases the perspective of the large-scale production of extracts and compounds for clinical trials. In addition, plant extracts have demonstrated promising synergistic effects when used in conjunction with existing antibiotic drugs to combat several types of bacteria, including some resistant strains. Clinical trials using plant extracts combined with other oral medications, in the various existing formulations, are needed to better understand their pharmacokinetics, pharmacodynamics and therapeutic potential. In a broader scenario, the development of new antiviral and antibacterial drugs can benefit a significant number of people affected by several viral and infectious diseases, many of which are endemic in various regions of the world.

## Figures and Tables

**Table 1:** Scope of the activity of some plants against resistant bacteria.

Plant Species [Botanical Family]	Active Against	Scope of Activity	Reference
*Achyranthes aspera* [Amaranthaceae]	MRSA ATCC 43300	IZ 6.3 ± 0.6 mm; MIC 42.0 ± 14.4 mg/mL	[50]
	MRPA ATCC 27853	IZ 6.2 ± 0.3 mm; MIC 200.0 ± 0.0 mg/mL	
	MRKP ATCC 00603	IZ 6.0 ± 0.0 mm; 50.0 ± 0.0 mg/mL	
*Acokathera oppositifolia* [Apocynaceae]	MRKP ATCC 33495	MIC 6.25 ± 0.0 mg/mL	[51]
*Ageratina adenophora* [Compositae]	MRSA ATCC 25923	IZ 10 ± 0.0 mm; MIC 12.5 mg/mL	[52]
*Areca catechu* [Arecaceae]	MRPA CCARM 2092	IZ 6.4 ± 0.5–16.3 ± 1.5 mm; MIC 5.6 µg/mL	[52]
	MRAB CCARM 12005	IZ 6.0 ± 0.0–17.7 ± 1.2 mm; MIC 5.6 µg/mL	
*Artemesia vulgaris* [Compositae]	MRSA ATCC 25923	IZ 10 ± 0.1 mm; MIC 12.5 mg/mL	[53]
*Azadirachta indica* [Meliaceae]	MRSA ATCC 43300	IZ 6.2 ± 0.3 mm; MIC 33.3 ± 14.4 mg/mL	[50]
	MRPA ATCC 27853	IZ 6.4 ± 0.4 mm; MIC 50.0 ± 0.0 mg/mL	
	MRKP ATCC 00603	IZ 6.1 ± 0.2 mm; MIC 41.7 ± 144 mg/mL	
*Cirsium englerianum* [Asteraceae]	MRSA ATCC 25923	IZ 28 ± 0.04 mm; MIC 16 μg/mL	[53]
*Euphorbia depauperata* *[Euphorbiaceae]*	MRSA ATCC 25923	IZ 26 ± 0.02 mm; MIC 4 μg/mL	[53]
*Hydrastis canadensis*. [Ranunculaceae]	MRSA AH1677	MIC 75 µg/mL	[54]
*Kalanchoe fedtschenkoi* [Crassulaceae]	MRAB CDC0033	MIC 256 μg/mL	[55]
	MREC CDC08	MIC > 256 μg/mL	
*Lawsoniainermis* [Lythracea]	MRSA ATCC 43300	IZ 15.5 ± 0.5 mm; MIC 4.2 ± 2.0 mg/mL	[50]
	MRPA ATCC 27853	IZ 12.5 ± 0.5 mm; MIC 4.2 ± 1.8 mg/mL	
	MRKP ATCC 00603	IZ 7.6 ± 0.5 mm; MIC 12.5 ± 0.0 mg/mL	
*Lippia adoensis* [Verbenaceae]	MRSA ATCC 25923	IZ 27 ± 0.56 mm; MIC 64 μg/mL	[49]
*Lippia javanica* [Verbenaceae]	MRPA ATCC 9721	MIC 6.25 ± 3.2 mg/mL	[49]
*Matricaria chamomilla* [Asteraceae]	MRSA ATCC 43300	IZ 30 ± 2 mm; MIC 0.781 mg/mL	[57]
	MRPA ATCC 27853	IZ 13.66 ± 1.52 mm; MIC 0.590 mg/mL	[57]
*Morella kandtiana* [Myricaceae]	MRAB CDC 0033	MIC > 256 μg/mL	[58]
	MBKP CDC 0076	MIC 256 μg/mL	
*Mentha* sp [Lamiaceae]	MRAB CI	MIC > 2 mg/mL	[59]
	MRKP CI	MIC >2 mg/mL	
	MRPA CI	MIC 2 mg/mL	
*Ocimun basilicum* [Lamiaceae]	MRAB CI	MIC > 2 mg/mL	[59]
	MRKP CI	MIC > 2 mg/mL	
	MRPA CI	MIC > 2 mg/mL	
*Oxalis corniculata* [Oxalidaceae]	MRKP CDC 0076	IZ 11 ± 0.0 mm; MIC 25 mg/mL	[53]
*Plectranthus barbatus* [Lamiaceae]	MRAB CI	MIC > 2 mg/mL	[59]
	MRKP CI	MIC 1 mg/mL	
	MRPA CI	MIC 2 mg/mL	
*Punica granatum* [Punicaceae]	MRKP CDC 0076	IZ 19–45 ± 0.7 mm	[60]
*Salvia triloba* [Lamiaceae]	MRSA ATCC 6538 P	IZ 9.5 mm	[61]
*Scutellaria barbata* [Lamiaceae]	MRAB CDC 0033	IZ 14–18 ± 0.0 mm; MIC 6.4 mg/mL	[62]
*Thymus zygis* L. [Lamiaceae]	MRSA ATCC 43300	IZ 75 ± 00 mm; MIC 02 ± 0.0009 μL/mL	[63]
	MRAB CDC 0033	IZ 71.5 ± 0.1 mm; MIC 02 ± 0.001 μL/mL	
*Thymus willdenowii* [Lamiaceae]	MRSA ATCC 43300	IZ 33 ± 0.2 mm; MIC 04 ± 00 μL/mL	[63]
	MRAB CDC 0033	IZ 30 ± 00 mm; MIC 04 ± 0.001 μL/mL	
*Zanthoxylum chalybeum* [Rutaceae]	MRSA ATCC 1677	MIC 16 μg/mL	[58]
	MREF ATCC 0044	MIC 32 μg/mL	

MRPA: multidrug-resistant *P. aeruginosa*; MRAB: multidrug-resistant *A. baumannii*; MRE: multidrug-resistant *Enterobacter* ssp.; MRSA: multidrug-resistant *S. aureus*; MREF: multidrug-resistant *E. faecium*; MRKP: multidrug-resistant *K. pneumoniae;* IZ: inhibition zone; MIC: minimal inhibitory concentration; CCARM: Collection of Cultures of Antimicrobial Resistant Microbes; CDC: Centers for Disease Control and Prevention; ATCC: American Type Culture Collection; CI: clinical isolates.

**Table 2:** Synergy effect of plant species with antibiotics towards bacterial pathogens.

Target Pathogen	Plant Species	Synergy Effect	Reference
*Aggregatibacter actinomycetemcomitans*	*Salvadora persica*	More than doubled the activity combined with metronidazole	[68]
*B. cereus*, *S. aureus*, *E. coli*, and *P. aeruginosa*	*Ficus nitida*	Antibacterial activity was enhanced in the presence of tetracycline	[69]
*E. coli* and *K. pneumoniae*	*Centaurea damascena*	Synergetic effect combined with gentamicin (ineffective for *E. coli*), vancomycin, ampicillin and chloramphenicol (ineffective for *K. pneumoniae*)	[70]
MDRAB and MDRPsA	*Pithecellobium clypearia*	Synergistic effect with imipenem and tetracycline ^a^	[71]
MDRPsA	*Coriandrum sativum*	Synergism in the presence of antibiotics including mezlocillin, cefoperazone, cefotaxime and levofloxacin	[72]
MRSA 1485279	*Vernonia condensata*	High MIC reduction combined with ampicillin ^a^	[73]
Multidrug-resistant enteric bacteria	*Carum copticum*	Reduced up to 64-fold MIC against *E. coli* with ciprofloxacin	[74]
*P. mirabilis*	*Petalostigma* spp.	Synergistic activity with penicillin-G, chloramphenicol and erythromycin	[41]
*S. aureus* ATCC 12600	*Origanum vulgare* and *Hypericum perforatum*	Combined extracts (1:1) increased inhibition over 3 times more than the individual extracts	[75]
*S. aureus* ATCC 25923 and *E. coli* ATTC 25922	*Vatica diospyroides*	Increased ampicillin efficacy; reduced the required antibiotic concentration by eight times	[76]
*S. aureus* strains 3993 and 4125	*Salvia officinalis*, *Senna macranthera*, and *Plectranthus ornatus*	Up to 8-fold reductions in the MIC, especially associated to ampicillin, kanamycin and gentamicin	[77]
*Treponema denticola*	*Cinnamomum zeylanicum*	More than doubled the activity combined with amoxicillin	[68]

MDRAB: multidrug-resistant *A.baumannii*; MDRPsA: multidrug-resistant *P. aeruginosa*; MRSA: methicillin-resistant *S. aureus*; MIC: minimum inhibitory concentration; ^a^ alteration in membrane permeability

**Table 4:** Traditional plant species that are promising in the search for new antiviral medicines.

Scope	Reference
**Indian Spices and Ayurvedic Herbs**	
Spices with anti-inflammatory properties with suggested beneficial action in the prevention and treatment of COVID-19 associated cytokine storm.	[116]
Spices useful for future design of new protease inhibitors effective against SARS-CoV-2.	[117]
Antiviral activities of spices, herbs, and derivatives, mechanisms of action, and prospects for future studies.	[118]
Mechanism of action of spices regularly used for cooking purpose to enhance the taste of food in India.	[98]
In silico evaluation of Indian traditional spices with medicinal properties for their inhibitory activity against SARS-CoV-2 spike proteins (SP) and main proteases (Mpro).	[119]
Immune impact of various Indian spices, potential to tackle the novel coronavirus, safety and toxicity aspects.	[120]
Traditional herbs used for protection against COVID-19 in North India.	[121]
Modulation of host immune responses by spice-derived bioactive components with protective immunity in COVID-19.	[122]
Preventive effect of Trikadu (mixture of *Zingiber officinale*, *Piper nigrum* and *Piper longum*) by action in the immune system.	[123]
Docking of gingerol, thymol, thymohydroquinone, cyclocurcumin, hydrazinocurcumin, components of Indian medicinal plants (ginger, black cumin, turmeric) against initially deposited spike structural proteins (PDB ID 6WPT) and mutant variant D-614G (PDB ID 6XS6).	[124]
Quick screening of traditional herbs/spices phytoconstituents by in silico study in polyherbal/Ayurvedic formulations.	[125]
**Indonesian herbal medicines**	
Several healthy drinks related to the COVID-19 pandemic.	[126]
**Tanzanian Traditional Medicine**	
Phytochemical screening of medicinal plants used to combat COVID-19 in Tanzania.	[127]
**Persian Traditional Medicine**	
New traditional Persian medicine-based drug, efficacy and safety assessment in COVID-19 patients with major symptoms.	[128]
**Other**	
Available and affordable complementary treatments for COVID-19.	[129]
Scientific evidence on potential role of spices in offering innate and adaptive immunity to human body.	[130]
Role of functional foods through modulating the host immune system and promoting the synthesis of agents effective against the coronavirus.	[131]
